# *EP300* contributes to high-altitude adaptation in Tibetans by regulating nitric oxide production

**DOI:** 10.24272/j.issn.2095-8137.2017.036

**Published:** 2017-05-18

**Authors:** Wang-Shan Zheng, Yao-Xi He, Chao-Ying Cui, zhuluobu Ou, jiquzong De, Yi Peng, Cai-Juan Bai, jizhuoma Duo, galanzi Gong, ba Bian, makangzhuo Bai, Yong-Yue Pan, la Qu, min Kang, renyangji Ci, mayangji Bai, Wei Guo, la Yang, Hui Zhang, Xiao-Ming Zhang, Yong-Bo Guo, Shu-Hua Xu, Hua Chen, Sheng-Guo Zhao, Yuan Cai, Shi-Ming Liu, Tian-Yi Wu, Xue-Bin Qi, Bing Su

**Affiliations:** ^1^College of Animal Science and Technology, Gansu Agricultural University, Lanzhou Gansu 730070, China; ^2^State Key Laboratory of Genetic Resources and Evolution, Kunming Institute of Zoology, Chinese Academy of Sciences, Kunming Yunnan 650223, China; ^3^High Altitude Medical Research Center, School of Medicine, Tibetan University, Lhasa Tibet 850000, China; ^4^Kunming College of Life Science, University of Chinese Academy of Sciences, Kunming Yunnan 650204, China; ^5^Chinese Academy of Sciences Key Laboratory of Computational Biology, Max Planck Independent Research Group on Population Genomics, CAS-MPG Partner Institute for Computational Biology, Shanghai Institutes for Biological Sciences, Chinese Academy of Sciences, Shanghai 200031, China; ^6^Center for Computational Genomics, Beijing Institute of Genomics, Chinese Academy of Sciences, Beijing 100101, China; ^7^National Key Laboratory of High Altitude Medicine, High Altitude Medical Research Institute, Xining Qinghai 810012, China; ^8^School of Life Science and Technology, Shanghai Tech University, Shanghai 200031, China; ^9^Collaborative Innovation Center of Genetics and Development, Shanghai 200438, China

**Keywords:** Tibetans, High altitude, Hypoxia, *EP300*, Genetic adaptation, Nitric oxide

## Abstract

The genetic adaptation of Tibetans to high altitude hypoxia likely involves a group of genes in the hypoxic pathway, as suggested by earlier studies. To test the adaptive role of the previously reported candidate gene *EP300* (histone acetyltransferase p300), we conducted resequencing of a 108.9 kb gene region of *EP300* in 80 unrelated Tibetans. The allele-frequency and haplotype-based neutrality tests detected signals of positive Darwinian selection on *EP300* in Tibetans, with a group of variants showing allelic divergence between Tibetans and lowland reference populations, including Han Chinese, Europeans, and Africans. Functional prediction suggested the involvement of multiple *EP300* variants in gene expression regulation. More importantly, genetic association tests in 226 Tibetans indicated significant correlation of the adaptive *EP300* variants with blood nitric oxide (NO) concentration. Collectively, we propose that *EP300* harbors adaptive variants in Tibetans, which might contribute to high-altitude adaptation through regulating NO production.

## INTRODUCTION

Tibetans are well adapted to high-altitude environments, in which the key environmental stress is hypobaric hypoxia. Physiologically, Tibetans show blunted responses to high altitude hypoxia, with low pulmonary vasoconstrictor response and low hemoglobin concentration compared with lowlanders moving to high altitude (Wu & Kayser, 2006). Previous genetic studies have reported a group of genes that show deep genetic divergence between Tibetans and Han Chinese. These genes are involved in the hypoxic pathway and therefore likely play important roles in the genetic adaptation to high altitude hypoxia found in Tibetans (Beall et al., 2010; Bigham et al., 2010; Peng et al., 2011; Simonson et al., 2010; Wang et al., 2011; Xu et al., 2011; Yi et al., 2010).

Hypoxia inducible factor 2α *(HIF2α*, also called *EPAS1*) and its negative regulator *EGLN1* are considered key genes responsible for Tibetan adaptation (Lorenzo et al., 2014; Peng et al., 2017; Xiang et al., 2013). Compared with these two genes, other reported candidate genes show relatively less between-population divergence, implying that they are probably modifiers for high-altitude adaptation. One reported example is heme oxygenase-2 (*HMOX2*), with Tibetan-enriched adaptive mutations of *HMOX2* shown to cause more efficient breakdown of heme during hemoglobin metabolism (Yang et al., 2016). However, the functional roles of other modifier genes are unknown. In addition, although we have a fundamental understanding of the genetic basis for Tibetan adaptation to high altitude, the studied genes thus far only explain a small part of the adaptive traits in Tibetans, highlighting the need for further genetic studies.

In reported genome-wide comparisons between Tibetans and Han Chinese, histone acetyltransferase p300 (*EP300*) is among the candidate genes showing signals of selection (Peng et al., 2011). *EP300* is located on human chromosome 22 (22q13.2), spanning about 88.9 kb with 31 exons (Eckner et al., 1994). It functions as a histone acetyltransferase regulating the transcription of genes by chromatin remodeling, and plays an essential role in regulating cell growth and division and promoting cell maturation and differentiation (Goodman & Smolik, 2000; Ogryzko et al., 1996).*EP300* is also a hypoxia switch, regulating hypoxia inducible factor 1α (*HIF1α*) transactivation through specific recognition and hydroxylation of asparagine (Anokhina & Buravkova, 2010; Liao & Johnson, 2007; Peng et al., 2011; Teufel et al., 2007). More importantly, *EP300* plays a role in the stimulation of hypoxia-induced genes, such as vascular endothelial growth factor (*VEGF*) (Gray et al., 2005; Teufel et al., 2007; Zhang et al., 2013). Furthermore, disruption of *EP300* function can cause Rubinstein-Taybi syndrome, a condition characterized by short stature, moderate to severe intellectual disability, distinctive facial features, and broad thumbs and first toes, an indication of its functional importance (Negri et al., 2016; Solomon et al., 2015; Teufel et al., 2007).

To understand the potential role of *EP300* in Tibetan adaptation to high altitude hypoxia, we resequenced the entire genomic region of *EP300*. Neutrality tests suggested a signal of positive Darwinian selection on *EP300* in Tibetans. Genetic association analysis indicated the involvement of *EP300* in regulating nitric oxide production.

## MATERIALS AND METHODS

### Tibetan samples and *EP300* resequencing

We resequenced a 108.9 kb genomic fragment of 47 unrelated Tibetan individuals, with sample details reported in previous study (Peng et al., 2011). We also obtained sequence data of the same gene region of 33 Tibetans from previously published genome sequencing (Lu et al., 2016). In total, we had sequencing data from 80 unrelated Tibetans.

### Selection tests of candidate variants

From the resequencing data (108.9 kb) of 80 Tibetans, we obtained 250 sequence variants. For quality control, we removed variants showing a significant deviation from the Hardy-Weinberg Equilibrium (HWE < 0.000 1) and variants with an excessive missing genotype rate (MGR > 0.05). A total of 185 variants remained after the filtering process. Following the methodology of Weir & Cockerham (1984), locus specific *F*_ST_ was calculated between Tibetans and the three lowland reference populations from the 1000 Genomes Project, which included 103 Han Chinese (CHB), 99 Europeans (CEU), and 108 Africans (YRI). Tajima's *D*-test was also performed to detect selection (Tajima, 1989).

For haplotype-based selection tests, the iHS score was calculated for each variant in Tibetans using selscan (Szpiech & Hernandez, 2014) based on the phased haplotypes, and only loci whose ancestral alleles were known with certainty were included (Voight et al., 2006). Additionally, XP-EHH analysis was used to detect the extended haplotypes resulting from positive selection (Sabeti et al., 2007). We computed XP-EHH scores using selscan (Szpiech & Hernandez, 2014) based on phased haplotypes of Tibetans and Han Chinese (reference population). The XP-EHH score of each variant was standardized by the mean XP-EHH and the standard deviation over the entire genome.

### Functional prediction and expression quantitative trait loci (eQTL) analysis of *EP300* candidate SNPs

Functional enrichment analyses of the candidate variants were performed using the Combined Annotation Dependent Depletion (CADD) database (http://krishna.gs.washington.edu/download/CADD/v1.3/1000G_phase3_inclAnno.tsv.gz), which incorporates data from ENCODE and NIH Roadmap Epigenomics using ChromHMM (https://sites.google.com/site/anshulkundaje/projects/epigenomeroadmap#TOC-Core-Integrative-chromatin-state-maps-127-Epigenomes-) (Ernst & Kellis, 2012).

We measured the evolutionary constraints of each variant using Genome Evolutionary Rate Profiling (GERP) (http://mendel.stanford.edu/SidowLab/downloads/gerp/). The GERP++ method was used to calculate site-specific RS scores and discover evolutionarily constrained elements (Davydov et al., 2010). Positive scores suggest evolutionary constraint, with higher scores indicating higher levels of evolutionary constraint.

The H3K4Me1 value indicates the maximum ENCODE H3K4 methylation level (maximum value observed across 16 ENCODE cell lines at a given position), where modification of histone proteins is suggestive of an enhancer and, to a lesser extent, other regulatory activities. The H3K4Me3 value indicates the maximum ENCODE H3K4 trimethylation level (maximum value observed across 16 ENCODE cell lines at a given position), where modification of histone proteins is suggestive of a promoter. The DNase-I hypersensitivity sites indicate chromatin regions hypersensitive to cutting by the DNase enzyme. In general, gene regulatory regions tend to be DNase-sensitive, and promoters are particularly DNase-sensitive. DNase-P indicates the *P*-value (PHRED-scale) of DNase evidence for open chromatin. The transcription factor binding site (TFBS) is indicated by the number of different overlapping ChIP transcription factor binding sites. It also defines the boundaries between active and heterochromatic DNA. Transcriptional repressor CTCF is a versatile transcription regulator involved in regulating the 3D structure of chromatin. In addition, splice site analysis indicates whether the tested variants are located in the ACCEPTOR or DONOR sequences.

The eQTL analysis for candidate *EP300* single nucleotide polymorphisms (SNPs) was conducted using publicly available datasets (Blood eQTL Browser: http://genenetwork.nl/bloodeqtlbrowser/).

### Measurements of physiological traits

Physiological data and blood samples were collected from 226 unrelated Tibetans permanently residing in Bange County (*n*=127, 37.41±3.8 years old) at an elevation of 4 700 m and Lhasa city (*n*=99, 35.33±6.8 years old) at an elevation of 3 600 m. Written informed consent was obtained from all participants. For physiological parameters, we determined the hemoglobin (Hb) concentration, arterial oxygen saturation (SaO_2_) level, and blood nitric oxide (NO) concentration, which are key adaptive physiological traits in Tibetans (Wu & Kayser, 2006).

The Hb concentration was measured using a HemoCue Hb 201+ analyzer (Angelholm, Sweden) and SaO_2_ was measured from the forefinger tip with a hand-held pulse oximeter (Nellcor NPB-40, CA, USA) at rest. Venous blood was collected for Hb measurement and DNA extraction. To reveal the NO level in serum, predominant species NO^2−^ and NO^3−^ were measured using a nitric oxide analyzer (Sievers Model-280, GE Analytical Instruments, Boulder, CO, USA).

### Genotyping and association analysis

We genotyped six candidate variants and conducted association analysis in 226 Tibetans. The variants were rs58268766, rs2076578, rs2076580, rs5758251, rs5758256, and rs2143694. Genotyping was conducted by the SNaPshot method on an ABI 3130 sequencer (Applied Bio-systems, Forster City, CA, USA). Genetic association analysis was conducted using PLINK 1.07 (Purcell et al., 2007). An additive genetic model was used because all tested variants were located in the non-coding region of *EP300* and likely influenced gene expression. For multiple test correction, we performed 100 000 permutations.

## RESULTS

### Resequencing of *EP300* in Tibetans and neutrality tests

We resequenced a 108.9 kb *EP300* genomic region, covering the 88.9 kb gene region as well as two 10 kb flanking regions upstream and downstream of *EP300*. In total, we obtained *EP300* sequencing data of 80 unrelated Tibetans.

**Figure 1:**
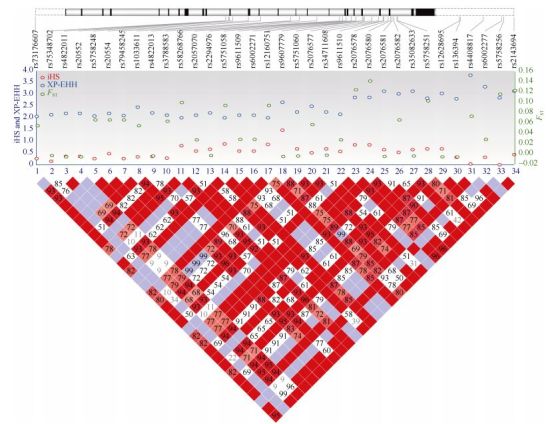
Information on 34 *EP300* candidate variants

We identified a total of 185 *EP300* sequence variants among the 80 unrelated Tibetans. After comparing with three lowlander populations, including Han Chinese, Europeans, and Africans, there were 149 shared variants. The remaining 36 variants were all rare mutations in Tibetans ( < 1.0%). To detect selection signals of these variants, we performed neutrality tests, including both allele-frequency-based (*F*_ST_ and Tajima's *D*) and haplotype-based tests (iHS and XP-EHH). Consistent with previous research (Peng et al., 2011), we observed many variants showing above-genome-average divergence (*F*_ST_ > 0.03) between Tibetans and Han Chinese. The highest *F*_ST_ was 0.14 for rs2076580. The derived allele at rs2076580 was predominant in Tibetans (76%), much higher than the frequencies in the lowland reference populations (48% in Han Chinese, 34% in Europeans, and only 3% in Africans). Consistently, in the haplotype-based XP-EHH test, we found 34 *EP300* variants showing high scores (XP-EHH > 0.2) (Supplementary Table S1). These high-XP-EHH variants were likely under positive Darwinian selection, and were located in a LD block spanning about 6 kb from intron-6 to the 3' flanking region (Figure 1). The allele-frequency-based Tajima's *D-*test was not significant, likely due to its insensitivity to recent selection. Overall, compared with the reported strong selection on *EPAS1* and *EGLN1* (Peng et al., 2011; Xiang et al., 2013; Xu et al., 2011), the selection on *EP300* in Tibetans was relatively weak, consistent with a modifier role in genetic adaptation to high altitude.

### Functional prediction and eQTL analysis of candidate *EP300* variants

With the detected signal of selection on *EP300* in Tibetans, the next question was what were the functional consequences of the variants under selection? We chose 34 candidate variants that showed high XP-EHH scores ( > 2.0), and except for two synonymous variants, most were non-coding. We performed functional prediction based on sequence conservation (GERP), transcription factor binding sites (TFBS), splicing motif, H3K4Me1/H3K4Me3 sites, and DNAase-I hypersensitive sites. There were 14 variants showing conserved sequences across species (GREP++ > 5.0), an implication of functional constraint. In addition, multiple variants were located in the H3K4Me1/ H3K4Me3 sites, suggesting their potential involvement in enhancer or promoter activities (Supplementary Table S1).

We also performed eQTL analysis using published data (Blood eQTL Browser: http://genenetwork.nl/bloodeqtlbrowser/), and found that three candidate variants showed highly significant association with the expression of *EP300* in blood (*P*=1.64×10^-54^ for rs2076578, *P*=3.63×10^-54^ for rs575825, and *P*=3.26×10^-54^ for rs2143694), suggesting that these variants are probably involved in the expression regulation of *EP300*. However, further experiments are needed to test their suggestive functions.

### Genetic association analysis of candidate *EP300* variants with multiple physiological traits in Tibetans

**Figure 2:**
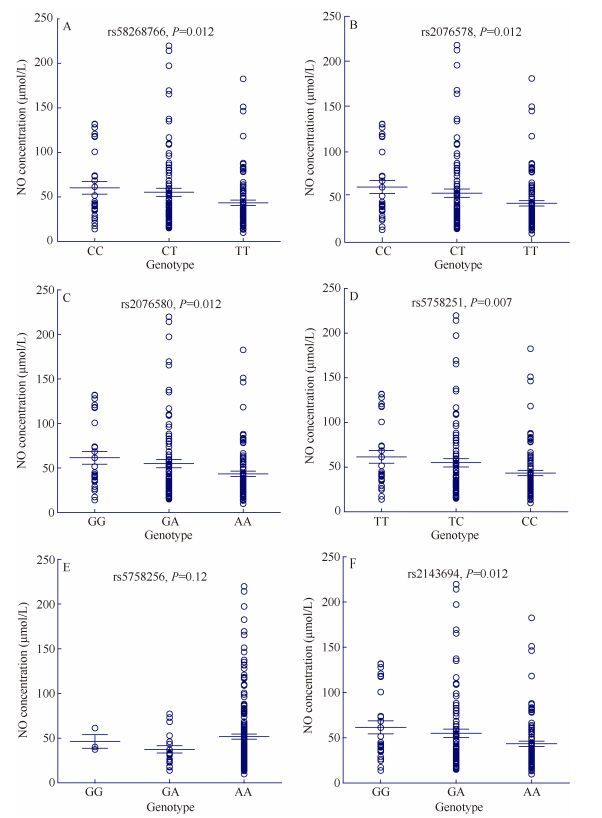
NO levels of different genotypes of six *EP300* variants in 226 Tibetans

To test whether the candidate *EP300* variants contributed to the adaptive traits in Tibetans, we collected data on three physiological parameters, including Hb, SaO_2_, and NO. A total of 226 unrelated adult Tibetans were included (91 males and 135 females from Lhasa and Bange, Tibetan Autonomous Region of China). Six candidate variants were selected based on their *F*_ST_ values and XP-EHH scores (*F*_ST_ > 0.1 and XP-EHH > 2.0). As shown in Table 1, five of the six variants showed significant association with blood NO level when an additive genetic model was assumed (*P* < 0.05, after multiple test corrections with permutations). The same result was observed when males and females were analyzed separately (Table 1), with no gender difference detected for average blood NO level. The presumably adaptive alleles were correlated with a decreased NO level and explained about 3% of NO variance. For example, the three genotypes at rs2076580 had NO levels of 61.53 μmol/L (GG genotype), 54.96 μmol/L (GA genotype), and 43.43 μmol/L (AA genotype), respectively, and each adaptive allele caused, on average, 15.8% decrease in NO in the blood (Figure 2). Hence, the *EP300* variants are likely involved in the regulation of blood NO production. In contrast, no association was detected for Hb or SaO_2_.

**Table 1:** Association of six *EP300* variants with three physiological traits in Tibetans

Trait	SNP ID	Male (*n*=91)		Female (*n*=135)		All (*n*=226)		
		Beta	EMP'	Beta	EMP'	Beta	EMP''	R^2^(%)
Hb	rs58268766	-1.83	0.63	3.16	0.25	0.63	0.86	7.24E-03
	rs2076578	-0.74	0.78	3.97	0.16	1.52	0.69	0.03
	rs2076580	-0.74	0.78	3.78	0.16	1.41	0.69	0.01
	rs5758251	-0.74	0.78	4.26	0.14	1.65	0.63	0.03
	rs5758256	8.59	0.24	-6.73	0.24	-1.11	1.00	3.26E-04
	rs2143694	-0.74	0.78	3.78	0.16	1.41	0.69	0.01
NO	rs58268766	-9.80	0.09	-9.06	0.06	-9.14	1.23E-02	2.98
	rs2076578	-10.00	0.08	-9.68	4.81E-02	-9.58	1.20E-02	3.13
	rs2076580	-10.00	0.08	-9.89	4.19E-02	-9.68	1.23E-02	3.21
	rs5758251	-10.00	0.08	-9.93	4.03E-02	-9.68	7.62E-03	3.20
	rs5758256	13.04	0.31	8.84	0.31	10.47	0.12	0.88
	rs2143694	-10.00	0.08	-9.89	4.19E-02	-9.68	1.23E-02	3.21
Sa0_2_	rs58268766	1.12	0.31	0.35	0.86	0.65	0.33	0.93
	rs2076578	0.94	0.38	0.21	1.00	0.51	0.40	0.67
	rs2076580	0.94	0.38	0.27	0.86	0.54	0.38	0.70
	rs5758251	0.94	0.38	0.30	0.86	0.56	0.38	0.72
	rs5758256	1.26	0.28	1.39	0.33	1.75	0.14	1.37
	rs2143694	0.94	0.38	0.27	0.86	0.54	0.38	0.70
Hb, hemoglobin concentration; NO, blood nitric oxide concentration; SaO_2_, blood oxygen saturation level. EMP', *P* value after multiple test corrections; EMP'', *P* value after multiple test corrections with sex as the covariant.								

## DISCUSSION

*EP300* is a candidate gene showing relatively deep allelic divergence between Tibetans and lowlanders (Peng et al., 2011). As previous data were obtained from DNA arrays with limited *EP300* variant coverage, whether *EP300* plays a role in Tibetan adaptation to high altitude has remained inconclusive. In this study, we resequenced the entire *EP300* gene region. In combination with published data, we showed that *EP300* in Tibetans has undergone positive Darwinian selection. *EP300* acts as a transcriptional coactivator of *HIF1α*, one of the most important hypoxic genes (Freedman et al., 2002; Gray et al., 2005; Lando et al., 2002). Functional prediction analysis suggested multiple *EP300* SNPs with potential functional effects. Hence, the function of selection on *EP300* is probably related to its role in the hypoxic pathway.

Importantly, we observed a significant association of many high XP-EHH variants with NO concentration. It has been proposed previously that high NO levels are an adaptive feature of Tibetans for high altitude living. Prior studies have shown that the NO levels of 88 Tibetans living at 4 300 m elevation were 10 times higher than those of 50 European-Americans living at 203 m elevation (Beall, 2007; Levett et al., 2011). High NO levels would allow for better vasodilation and therefore better blood flow (Beall, 2007), which is an adaptive physiological trait observed in Tibetans. Enzymes eNOS and iNOS synthesize NO products in the body. They are encoded by *NOS3* and *NOS2*, respectively, and both contain hypoxia-response-element (HRE) motifs in the gene promoter regions (Coulet et al., 2003; Melillo et al., 1995). In other words, *NOS3* and *NOS2* can be directly regulated by *HIF1*α and *HIF2*α. Therefore, the observed association of *EP300* with blood NO level can be explained by the co-transactivating role of *EP300* in expression regulation of *HIF1*α and eventually its downstream genes, including *NOS3* and *NOS2*. Notably, eNOS mainly functions in blood vessels (Matouk & Marsden, 2008). As *EP300* works in histone modification (Goodman & Smolik, 2000; Ogryzko et al., 1996), whether the functional role of *EP300* in high-altitude adaptation involves downstream gene regulation through chromatin remodeling is yet to be tested.

Under hypoxic conditions, in addition to the pathway of stabilizing HIF with reduced PHD2 hydroxylation, the nitroso sulfation of the HIF element is also important (Foster et al., 2003), and NO is the key component of nitroso sulfation. It has been reported that hypoxia upregulates iNOS, and thereby increases NO products in tissues and cells, especially in the case of chronic hypoxia. NO helps create a blunted response to hypoxia by inhibiting the oxygen consumption of mitochondria and consequently provide more oxygen for PHDs to reduce the levels of HIF proteins caused by hypoxia (Won et al., 2007). Collectively, NO is not only involved in the process of blood flow control, but is also a regulator of blood oxygen utilization (Ho et al., 2012). As shown in our results, the adaptive alleles were associated with a decreased level of blood NO, which might serve as a protection measure for Tibetans from overproduction of NO, and might be similar to the relatively low hemoglobin concentrations observed in Tibetans (Beall et al., 2010).

In summary, we proved that *EP300* has been under positive Darwinian selection, with a significant association with blood NO levels in Tibetans. These data suggest that *EP300* likely contributes to Tibetan adaptation to high-altitude. However, further studies are needed to reveal the underlying molecular mechanism.

## ACKNOWLEDGEMENTS

We are grateful to all the volunteers participated in this study.
